# A Synthetic Nanoparticle Based Vaccine Approach Targeting MSP4/5 Is Immunogenic and Induces Moderate Protection Against Murine Blood-Stage Malaria

**DOI:** 10.3389/fimmu.2019.00331

**Published:** 2019-03-15

**Authors:** Kirsty L. Wilson, Dodie Pouniotis, Jennifer Hanley, Sue D. Xiang, Charles Ma, Ross L. Coppel, Magdalena Plebanski

**Affiliations:** ^1^Department of Immunology and Pathology, Faculty of Medicine, Nursing and Health Sciences, Central Clinical School, Monash University, Melbourne, VIC, Australia; ^2^School of Health and Biomedical Sciences, RMIT University, Bundoora, VIC, Australia; ^3^Department of Microbiology, Monash University, Clayton, VIC, Australia

**Keywords:** blood-stage, malaria, nanoparticles, adjuvant, immunogenicity, protection

## Abstract

Malaria remains a significant health problem in many tropical and sub-tropical regions. The development of vaccines against the clinically active blood-stage of infection needs to consider variability and polymorphism in target antigens, and an adjuvant system able to induce broad spectrum immunity comprising both antibodies and helper T cells. Moreover, recent studies have shown some conventional pro-inflammatory adjuvants can also promote expansion of immunosuppressive regulatory T cells (Treg) and myeloid derived suppressor cells (MDSC), both of which could negatively impact malaria disease progression. Herein, we explore the ability of a model nanoparticle delivery system (polystyrene nanoparticles; PSNPs), previously proven to not induce conventional inflammation, Treg or MDSC, to induce immunity to MSP4/5 from *Plasmodium yoelii*, a member of the MSP4 and MSP5 family of proteins which are highly conserved across diverse malaria species including *P. falciparum*. The results show PSNPs-MSP4/5 conjugates are highly immunogenic, inducing immune responses comprising both T helper 1 (Th1) and Th2 cellular immunity, and a spectrum of antibody subclasses including IgG1, IgG2a, and IgG2b. Benchmarked against Alum and Complete Freund's Adjuvant (CFA), the immune responses that were induced were of comparable or higher magnitude, for both T cell frequencies by ELISpot and antibody responses in terms of ELISA end titer. Importantly, immunization with PSNPs-MSP4/5 induced partial protection against malaria blood-stage infection (50–80%) shown to be mechanistically dependent on interferon gamma (IFN-γ) production. These results expand the scope of adjuvants considered for malaria blood-stage vaccine development to those that do not use conventional adjuvant pathways and emphasizes the critical role of cellular immunity and specifically IFN-γ producing cells in providing moderate protection against blood-stage malaria comparable to Freunds adjuvant.

## Introduction

Malaria remains a global health problem, affecting over 200 million people annually with ~40,000 deaths (1). It is caused by the plasmodium parasite, of which *Plasmodium falciparum* (*P. falciparum*) and *Plasmodium vivax (P. vivax)* are the most widespread. There is an urgent need for the development of an effective and long-lasting malaria vaccine. The most advanced pre-erythrocytic stage vaccine RTS, S provides <40% protection against disease in African populations (1, 2). Since any break-through parasites progressing to the blood-stage sustain transmission, pre-erythrocytic vaccines alone may be insufficient to support complete protection or eradication. Additionally, the blood-stage of malaria is symptomatic of disease, highlighting the need to eradicate parasites at or before this stage.

One of the major challenges to designing an effective vaccine for malaria is the identification of appropriate antigen targets. Many candidates are polymorphic or poorly immunogenic, thus making them inadequate vaccine antigens. One group of potential blood-stage vaccine antigens are the merozoite surface proteins (MSP) (3). MSP1, MSP2, and MSP3 have been used in human vaccine trials alone or in combination vaccines (4–6) but demonstrate little protective efficacy (6). Similar results have been seen with other leading blood-stage proteins from the merozoite, such as AMA1 which has shown promising humoral immunogenicity (7, 8) but only modest protective efficacy (9). However, there have been very few trials assessing efficacy in humans, particularly in children for blood-stage vaccines and many more are needed. Vaccine development at this stage mainly focuses on generating a neutralizing antibody response to prevent erythrocyte invasion (similar to that seen with naturally acquired immunity) though inducing strong cellular responses may be just as important to promote protection. Therefore, it is important to identify the antigenic epitopes that are not only highly immunogenic but also protective.

MSP4 and MSP5 are candidates for blood-stage vaccines as they show limited antigenic diversity in both *P. falciparum* (10–12) and *P. vivax* (13, 14). MSP5 is largely conserved across both these strains, and also geographic locations of Plasmodium (15), with polymorphisms detected only in specific gene regions (16). Naturally acquired antibodies have been detected to MSP4 (17, 18) and high antibody levels have been associated with a protective role in subsequent malaria seasons (18), though the precise cause of protection in these cohorts is yet to be fully defined. Similarly, naturally acquired antibodies against MSP4 are associated with reduced clinical malaria cases (19) and low level frequency of cross reactive antibodies have been detected to both *P. falciparum* and *P. vivax* (20). Though possibly attributed to anti-MSP antibody production, the exact correlates of protection in these studies are yet to be identified, although it does indicate these antigens are good candidates for inclusion in vaccines. Importantly, functional MSP5 specific T cell responses have also been observed to *P. falciparum* and *P. vivax* (21). The low antigen diversity of MSP4 and MSP5 and correlation with protection makes them attractive vaccine candidates compared to the large number of other highly polymorphic antigens. In animal studies, the murine equivalent homolog of MSP4 and MSP5, MSP4/5, has shown protection using different vaccine formulations (22–25), and protection is enhanced when administered in combination with MSP1 (26). Most of these studies examine antibodies in the protective response, with less known about the protective role of the cellular immune response. One study showed that the selected adjuvant AFCo1 (synthetic cochleate structures) enhanced both antibodies and T cell responses against MSP4/5, attributable to the induced Th1-like immune responses (27). Thus, it is important for vaccine design to not only aim to induce antibody responses, but also cell mediated immunity.

One possible reason for the low protective responses seen in recent malaria vaccine trials may be due in part to adjuvant or vaccine delivery platform selection. Optimal antigen and adjuvant combinations are imperative for vaccine efficacy. Alum is currently one of the only licensed adjuvants for widespread human use (28) and the RTS, S vaccine in humans contains the proprietary adjuvant AS01, consisting of liposomes, monophosphoryl lipid A (MPLA, the non toxic derivative of LPS) and the saponin QS-21 (29). Both these adjuvants, as well as most adjuvants in development for human use, are pro-inflammatory (inducing cytokines such as interleukin [IL-6] and/or tumor necrosis factor [TNF]) (30). Recent literature suggests that such pro-inflammatory adjuvants could also promote the subsequent expansion of regulatory T cells (Treg) and myeloid derived suppressor cells (MDSC), which may be a natural mechanism aimed to turn off excessive inflammation (31). This property may limit the persistence of immunity, as well as potentially increase the frequency of cells such as TNFR2+ Treg associated with the development of severe malaria (32). It is therefore useful to explore whether non-inflammatory adjuvants and vaccine carriers, which do not induce Treg or MDSC, can also induce malaria specific immunity of sufficient type and magnitude to protect against a malaria challenge.

Viral sized 40–50 nm polystyrene nanoparticles (PSNPs) are non-inflammatory and when used as adjuvanting antigen carriers in experimental vaccines, induce high levels of CD4+ and CD8+ T cells, as well as antibodies (33–37). Though there are many different types of nanoparticles currently being used for malaria vaccines (38, 39) [reviewed in Powles et al. (40)], PSNPs in this size range (and negatively charged) can target antigen presenting cells (APCs) in the local lymph nodes to increase uptake and presentation of the vaccine antigen, leading to long lasting protective immune responses in murine studies (33, 34, 36). These PSNPs have been shown to induce high level CD8+ T cell responses when covalently coupled with malaria liver stage peptide epitopes, however not when simply “mixing” the PSNPs with the peptide (31, 41). Moreover, they have been confirmed to be “inert” in terms of failing to activate inflammatory cytokine or MAPK/ERK mediated inflammatory pathways (37, 42) and do not induce TNFR2+ Treg or MDSC (31). These inert and biocompatible PSNPs have repeatedly shown to be safe and well-tolerated in numerous animal models at both low and high doses (33, 36, 37, 43, 44), and can be coupled to different protein and peptide antigens with high efficiency and loading capacities (31, 37, 44). Additionally, recent pre-clinical studies progressing the translation of this approach as a cancer vaccine have seen no toxic or inflammatory effects when injecting similar PSNP doses in mice for up to 4 times at weekly intervals (44). Whilst the exact clearance mechanism of these PSNPs is unknown, it is known that nanoparticles in this viral size range allow their excretion from the body via hepatobiliary elimination and ultimately via feces (45).

Herein we assess both the immunogenicity and protective efficacy of blood-stage malaria vaccines in an animal model of malaria, formulated using PSNP antigen carriers using MSP4/5 as the target antigen and compared to formulations with MSP4/5 adjuvanted with Alum or the experimental adjuvant Complete Freund's Adjuvant (CFA).

## Materials and Methods

### Mice

Six-to-eight-week-old BALB/c and IFN-γ gene knock out (KO) mice were purchased from the Austin Research Institute or Monash Animal Services. Austin Health and the Alfred Medical Research and Education Precinct (AMREP) Animal Ethics Committees approved the use of all animals and procedures.

### Recombinant MSP4/5 Production

Recombinant MSP4/5 generated in *E. coli*, described in Kedzierski et al. (22), was kindly provided by Professor R. Coppel for use in these studies. The protein concentration of recombinant MSP4/5 was determined by Bicinchoninic Assay (BCA, Thermo Fisher) following manufacturer's instructions.

### Conjugation of MSP4/5 to PSNPs

Conjugation of MSP4/5 to PSNPs was performed as described previously (35, 37). Briefly, 40–50 nm carboxylated polystyrene nanoparticles (PSNPs, Polysciences Inc, USA) at a final 1% solids (~1.9 × 10^14^ PSNPs/ml, unless otherwise stated) were pre-activated using a 2-*N*-morpholino-ethanesulfonicacid buffered (MES; 50 mM final, pH 6.2) solution of 1-ethyl-3-(3-dimethylaminopropryl) carbodiimide hydrochloride (EDC; 4 mg/ml final) (Sigma Aldrich) (with sulfo-NHS for some conjugations) for 15 minutes on a rotating wheel at room temperature (RT). MSP4/5 was added (target final concentration of 0.4 or 1 mg/ml) and further incubated for 2–3 hours (h) at RT. The conjugation reaction was stopped by the addition of glycine (7 mg/ml final) for a further 30 minutes at RT. Conjugation mixture was dialyzed using 100–300 kDa dialysis membrane overnight against phosphate buffered saline (PBS) at 4°C. Dialysis against 100–300 kDa cutoff membrane permits retention of the PSNPs conjugated to the antigen, and any remaining activating agents and unconjugated protein [MSP4/5 is ~36kDa (24)] are dialyzed out. This process also allows for buffer exchange into a physiological solution, such as PBS, for immunizations. Conjugated PSNPs were stored at 4°C and sonicated for 15 minutes before use to create a uniform and homogenous formulation for immunizations.

### Vaccine Formulations and Immunizations

Mice were immunized intradermally (id) once or twice with the following vaccine formulations: 100 μl of either PSNPs conjugated to MSP4/5 or MSP4/5 with adjuvant, 2–3 weeks apart for immunogenicity studies. Adjuvanted vaccine formulations contained either Alum (Rehydragel HPA, General Chemical) at 0.3 g/ml with MSP4/5 in PBS, or a prime with MSP4/5 emulsified in CFA at a 1:1 v/v ratio and boost with Incomplete Freund's Adjuvant (IFA) at a 1:1 v/v ratio. Ten to fourteen days following the last immunization, mice were humanely sacrificed by CO_2_ asphyxiation. Conjugated and adjuvanted MSP4/5 protein doses, immunization volume and route were matched per experiment, with specific vaccine formulations provided in each of the figure legends. For challenge experiments, mice were injected with parasitized red blood cells (pRBCs) 3 weeks following the last immunization (see detailed procedure below).

### ELISpot

Splenocytes from immunized animals were isolated from 10 to 14 days following the last immunization and assessed by ELISpot for IFN-γ and IL-4 production. Ninety six well multiscreen plates (MAHA, MAIP or MSIP, Millipore) were coated overnight at 4°C with 5 μg/ml anti-mouse IFN-γ (AN18, Mabtech) or anti-mouse IL-4 (BVD4-1d11, Mabtech or BD Biosciences). All wells were washed 5 times with PBS and blocked with RPMI 1640 (Gibco, Life technologies) containing 10% FBS (further supplemented with 100 units/ml penicillin, 100 μg/ml streptomycin, 4 mM L-glutamine, 1 M Hepes, and 0.1 mM 2- mercaptoethanol, to make complete media) for 2 h at 37°C. Splenocytes were added at a final concentration of 0.5 × 10^6^ or 1 × 10^6^ cells per well and co-incubated with recall antigens (MSP4/5 at 15–25 μg/ml final for each recall antigen) in complete media for 12–16 h at 37°C. Media alone and positive control Concanavalin A (1 μg/ml final) wells were also added. Following incubation, plates were washed with PBS (MSIP plates) or PBS containing 0.05% Tween 20 (MAHA and MAIP plates) and 1 μg/ml anti-mouse IFN-γ-biotin (R4-6A2, Mabtech) or anti-mouse IL-4-biotin (BVD6-24G2, Mabtech or BD Biosciences) in 0.5% FBS/PBS was added for 2 h at RT. Plates were washed and 1 μg/ml Streptavidin-ALP or extravidin-ALP in 0.5% FBS/PBS was added for a further 1–2 h at RT. Plates were given a final wash with PBS followed by reverse osmosis water and spots were developed using an AP colorimetric kit (Bio-Rad). Once plates were dry, spots were counted using an AID ELISpot Reader system (AutoImmune Diagnostika, GmbH, Germany).

### ELISA

Sera from immunized animals was collected at endpoint and assessed for antigen specific antibody production by ELISA. Ninety six well plates (polyvinyl chloride micro-titer plates, Costar, or Nunc Maxisorp plates) were coated with 5 μg/ml of MSP4/5 in carbonate/bicarbonate coating buffer overnight at 4°C. Plates were washed with 0.05% Tween 20/PBS and blocked with 2% bovine serum albumin (BSA)/PBS or 5% skim milk/PBS and incubated for 1 h at 37°C. Plates were washed as above and serial dilutions of sera added and incubated for 2 h at 37°C (or 4°C overnight). Plates were washed and horseradish peroxidase (HRP) conjugated anti-mouse total Ig or IgG antibodies (1/2000 dilution for total IgG/A/M, Zymed, and sheep anti-mouse IgG, Amersham Biosciences, UK) added for 1 h at 37°C. Where indicated HRP conjugated subclass antibodies were used as the secondary antibody and incubated for 1 h at 37°C (HRP-IgG1 at final 1/1,000, HRP-IgG2a at final 1/1,000 and IgG2b-biotin at final 1/250 dilution, BD Biosciences). Biotinylated secondary antibodies were followed with a further incubation of streptavidin-HRP (1/1,000, Amersham biosciences) for 1 h at 37°C. Plates were given a final wash and antibody detection was developed using ABTS or TMB substrate solution before being stopped with 1 M HCl. Absorbance was read at optical density (OD) 405–450 nm on a plate reader (Fluostar Optima, BMG lab technologies, or Multiscan GO, Thermo Fisher). Antibody end titers were calculated closest to the serum dilution at which the OD was equal to the mean of the naïve sera (averaged across naïve mice for all dilutions tested) plus 3 standard deviations.

### Malaria Infection Challenge

*Plasmodium yoelii* parasites were cultured *in vivo* in naïve mice and pRBCs harvested when mice reached a required level of parasitemia (~4–5%). pRBCs were harvested from infected mice and experimental mice were infected with 5 × 10^4^ or 5 × 10^5^ pRBCs in PBS by intraperitoneal injection (total volume 100 μl, 50 μl either side of abdomen). Blood parasitemia levels were monitored every 1-2 days beginning from day 3 until ~21–25 days post-infection. Blood parasitemia levels were calculated by venous blood smears that were Giemsa stained and counted using a light microscope. A representative field at 100X magnification was chosen and the number of pRBCs including all ring, merozoite, and schizont stages calculated compared to the total number of RBCs in the field. Mice were humanely euthanized when blood parasitemia levels reached >70%, according to ethical approval, and parasitemia levels and number of surviving mice (% survival) at each time point calculated.

### Statistics

Statistical analysis was performed as follows; One or two-way ANOVA with *post hoc* Tukey analysis was used to assess differences between groups for immunogenicity (ELISpot and ELISA) assays. Kaplan Meyer survival curves with log-rank (Mantel-Cox) test comparisons were used for survival analysis. All statistical analyses were performed using Graphpad Prism software (v.7.02) and statistical significance was set at *p* < 0.05. Data is expressed as mean ± standard deviation (SD) for each group, with group sizes and additional information indicated in the relevant figure legends.

## Results

### Highly Immunogenic Vaccine Formulations of PSNPs Conjugated to MSP4/5 (PSNPs-MSP4/5) Induce Broad Immune Responses Including Th1 and Th2 Cells and Multiple Antibody Subclasses

Identifying an immunogenic and highly conserved protein for blood-stage malaria remains a challenge for vaccine development. MSP4 and MSP5 are promising blood-stage vaccine candidates in humans, as they are relatively conserved in different strains of Plasmodium compared to other highly polymorphic antigens. The murine equivalent *P. yoelii* MSP4/5 has been identified as a protective blood-stage antigen in prior murine studies (23, 25, 46), therefore, we wanted to assess this protein using our nanoparticle delivery system.

**Figure 1:**
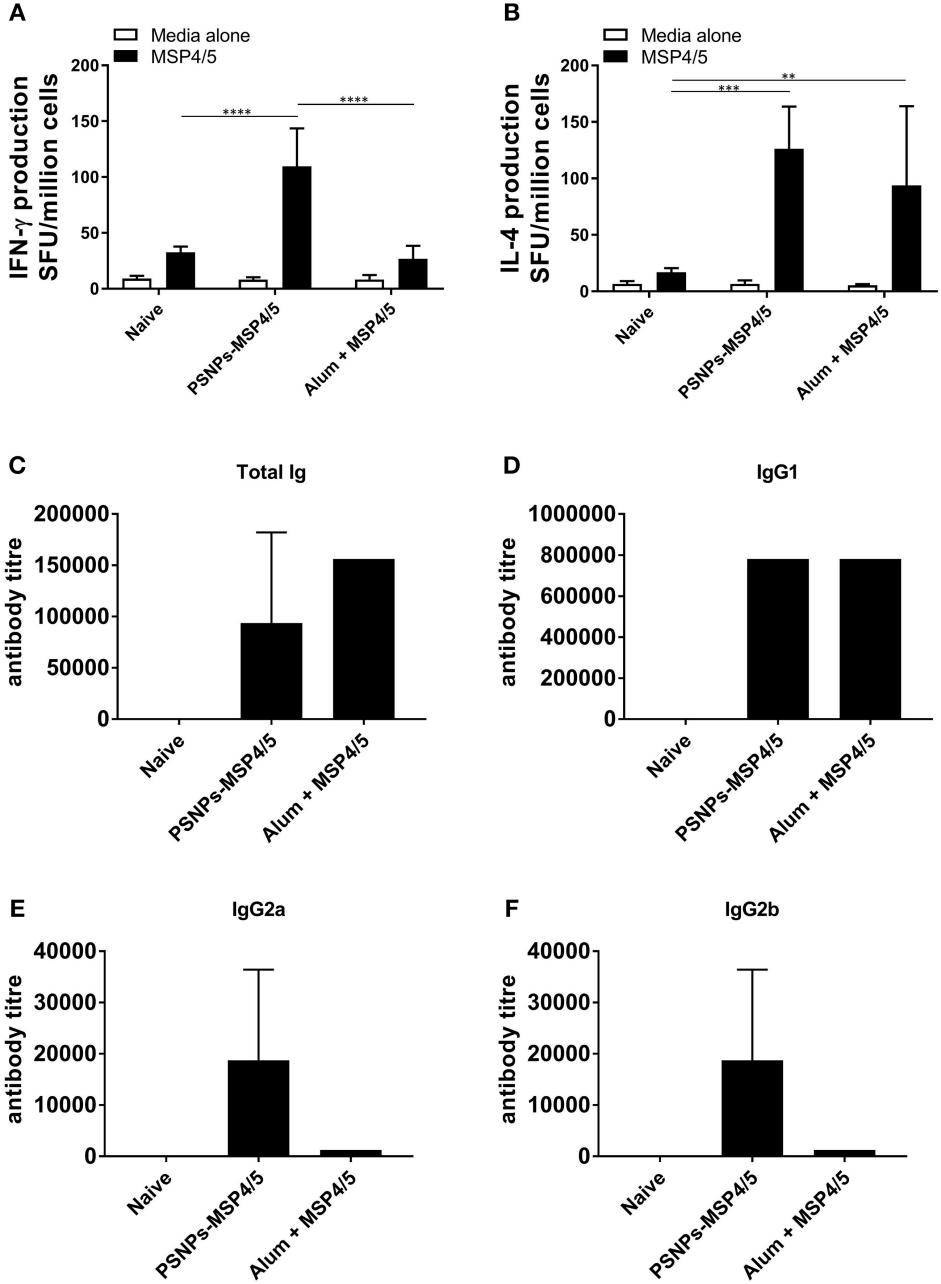
Diverse Th1 and Th2 associated immunogenicity induced by PSNPs-MSP4/5 compared to Alum adjuvanted vaccines. BALB/c mice were immunized twice intradermally at the base of tail (100 μl/immunization), 14 days apart with PBS (Naive), PSNPs conjugated to MSP4/5 (PSNPs-MSP4/5, 40 μg/ml) or MSP4/5 in Alum (Alum + MSP4/5, 0.3 g/ml Alum and 40 μg/ml MSP4/5 in PBS). Fourteen days after the last immunization, mice were humanely sacrificed and splenocytes and serum collected for immunogenicity analysis. **(A)** IFN-γ and **(B)** IL-4 producing T cells were assessed by ELISpot and **(C)** Total IgG/A/M, **(D)** IgG1, **(E)** IgG2a, and **(F)** IgG2b anti-MSP4/5 antibody levels were measured by ELISA. Data is shown as mean spot forming units (SFU) ± SD for ELISpot and mean antibody titer ± SD of duplicate measurements of pooled sera per group for ELISA. ^**^p < 0.01, ^***^p < 0.001, ^****^p < 0.0001 by two-way ANOVA with *post hoc* Tukey analysis. *n* = 4 mice per group.

To assess the immunogenicity of PSNPs-MSP4/5 we compared this vaccine to one containing MSP4/5 mixed with the most widely used adjuvant, Alum. BALB/c mice were immunized twice intradermally at the base of tail and 14 days following the last immunization T cell (from whole splenocytes) and serum antibody responses were measured by ELISpot and ELISA, respectively. PSNPs-MSP4/5 immunization elicited high levels of antigen specific IFN-γ producing T cells, significantly higher than mice immunized with Alum and naïve controls (Figure 1A, *p* < 0.0001). Whilst Alum did not induce significantly higher IFN-γ producing T cells above control conditions, both the PSNPs-MSP4/5 and Alum + MSP4/5 groups induced similar levels of IL-4 producing T cells compared to naïve mice (Figure 1B, *p* < 0.001, *p* < 0.01, respectively). Alum is known to induce Th2 biased responses which is reflected in the above results. Furthermore, when looking at total antibody levels induced by vaccination, both Alum and PSNPs induced comparable levels of total Ig (Figure 1C) and similar levels of IgG1 antibodies (Figure 1D), the latter being classically associated with a Th2 response. Mice immunized with PSNPs-MSP4/5 developed higher IgG2a and IgG2b antibodies compared to either the Alum + MSP4/5 or the naïve groups but these responses were not significant (Figures 1E, F). These results indicate PSNPs induce a spectrum of IgG antibody subclasses as well as both IFN-γ-producing Th1 and IL-4-producing Th2 cells.

### Comparable Immunogenicity of PSNPs-MSP4/5 Nanovaccines Against “Gold Standard” Experimental Vaccine Formulations of MSP4/5 in CFA/IFA

**Figure 2:**
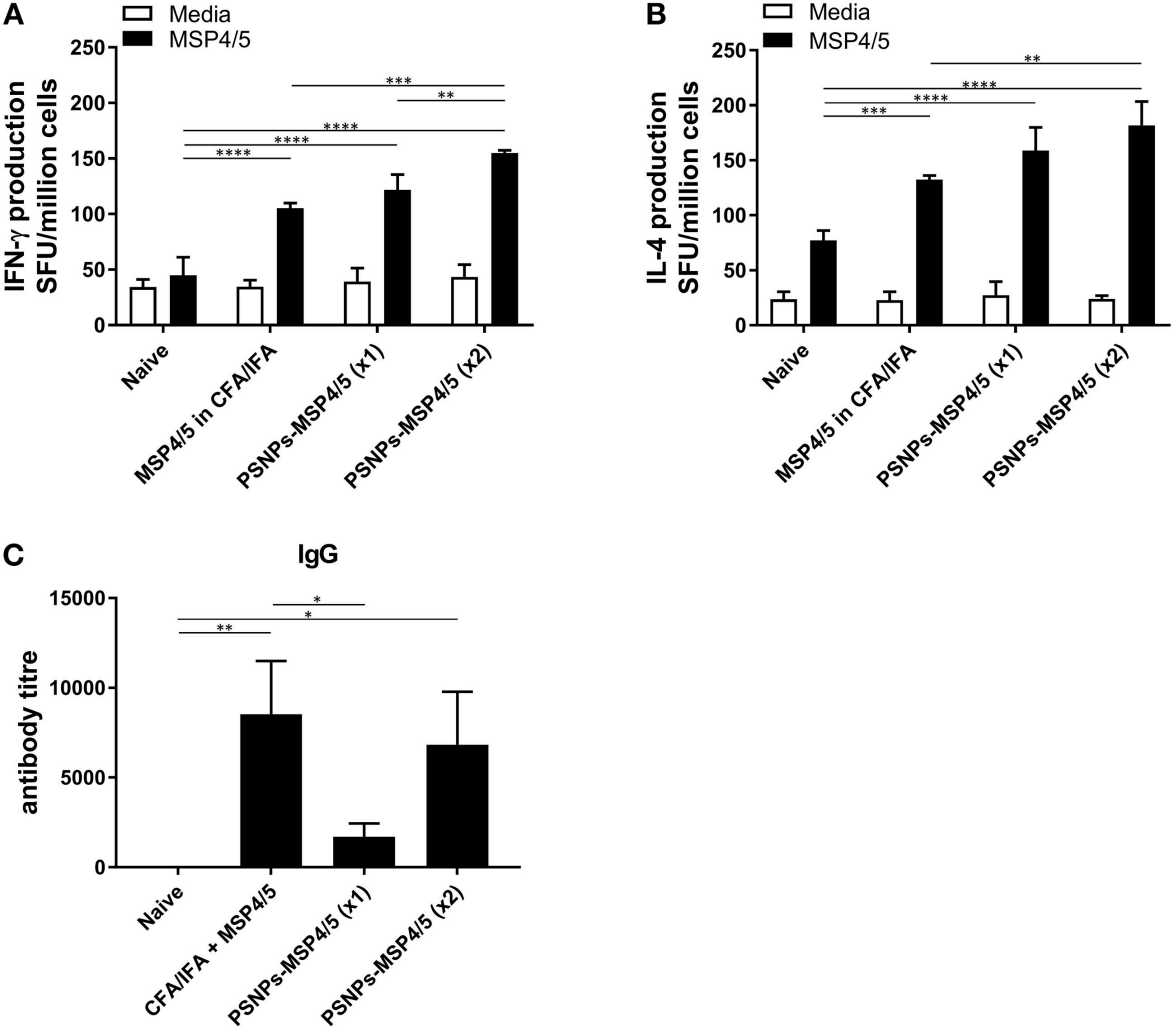
Immunogenicity of PSNPs-MSP4/5 is comparable to CFA/IFA adjuvanted vaccines. BALB/c mice were immunized intradermally (100 μl per immunization) with one (PSNPs-MSP4/5 x1) or two (PSNPs-MSP4/5 x2) immunizations of PSNPs-MSP4/5 (100 μg/ml), or MSP4/5 in CFA (100 μg/ml MSP4/5 mixed 1:1 v/v in CFA) followed by MSP4/5 in IFA (100 μg/ml MSP4/5 mixed 1:1 v/v in IFA), or twice with PBS alone (Naïve). Ten days after the last immunization, mice were humanely sacrificed and splenocytes and serum collected for immunogenicity analysis. **(A)** IFNγ and **(B)** IL-4 producing T cells were assessed by ELISpot assay and **(C)** Total IgG antibody levels were measured by ELISA. Data is shown as mean SFU ± SD per group for ELISpot and mean antibody titer ± SD per group for ELISA. ^*^p < 0.05, ^**^p < 0.01, ^***^p < 0.001, ^****^p < 0.0001 by two-way ANOVA for ELISPOT and one-way ANOVA for ELISA analyses. *n* = 6−8 mice per group.

The immune responses generated by PSNPs-MSP4/5 immunizations were comparatively similar for Th2, and better for Th1 type responses, compared to the conventional adjuvant Alum. Therefore, we further tested the robustness of these responses against the strong experimental Th1 inducing adjuvant, CFA, followed by a boost with IFA, and compared against one or two immunizations with PSNPs-MSP4/5. MSP4/5 in CFA/IFA induced functional IFN-γ and IL-4 producing T cells, as well as IgG antibodies, significantly higher than naïve mice (Figures 2A–C, *p* < 0.0001, *p* < 0.001, *p* < 0.01, respectively). One immunization with PSNPs-MSP4/5 was comparable to MSP4/5 in CFA/IFA for both IFN-γ and IL-4 producing T cell immune responses (Figures 2A,B), and significantly higher than naïve responses (Figures 2A,B, *p* < 0.0001). Two immunizations with PSNPs-MSP4/5 elicited the highest levels of IFN-γ and IL-4 T cell responses, significantly higher than all other groups for IFN-γ producing T cells (Figure 2A, *p* < 0.0001 compared to naïve, *p* < 0.001 compared to CFA/IFA and *p* < 0.01 compared to one immunization with PSNPs-MSP4/5) and significantly higher than MSP4/5 in CFA/IFA and naïve groups for IL-4 producing T cell responses (Figure 2B, *p* < 0.0001 compared to naïve and *p* < 0.01 compared to CFA/ IFA). Antibody titers were also different when comparing one vs. two immunizations with PSNPs-MSP4/5. Two immunizations of PSNPs-MSP4/5 elicited higher antibody titers compared to one immunization with PSNPs-MSP4/5 (Figure 2C, not significant) and comparable antibody titers to MSP4/5 in CFA/IFA (Figure 2C). MSP4/5 in CFA/IFA induced a significantly higher antibody end titre compared to only one immunization of PSNPs-MSP4/5 (Figure 2C, *p* < 0.05).

**Figure 3:**
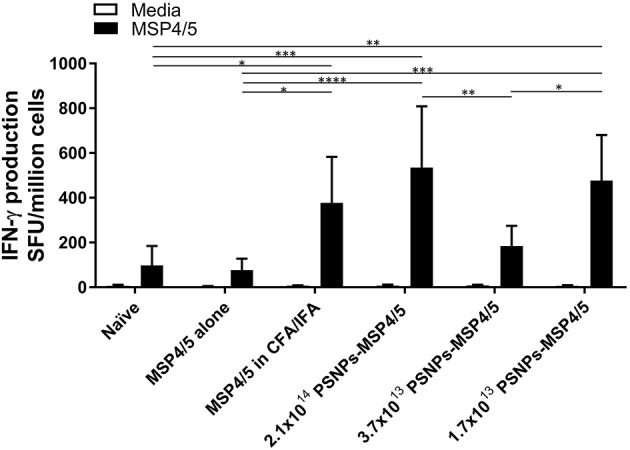
IFN-γ producing T cell responses induced by de-escalating doses of PSNPs compared to CFA/IFA. BALB/c mice were immunized intradermally at the base of tail (100 μl/immunization) and boosted 3 weeks later with PBS alone (Naïve), MSP4/5 alone (80 μg per mouse), MSP4/5 in CFA (80 μg per mouse, 1:1 v/v with CFA) for the initial immunization and MSP4/5 in IFA (80 μg per mouse, 1:1 v/v with IFA) for the boost, or 3 different doses of PSNPs conjugated to MSP4/5 (2.1 × 10^14^, 3.7 × 10^13^, 1.7 × 10^1^ PSNPs/ml, 80 μg MSP4/5 per mouse). Fourteen days after the last immunization, mice were humanely sacrificed and splenocytes and serum collected for immunogenicity analysis. IFN-γ producing T cells were assessed by ELISpot. Data is shown as mean SFU ± SD per group for ELISpot ^*^p < 0.05, ^**^p < 0.01, ^***^p < 0.001, ^****^p < 0.0001 by two-way ANOVA with *post hoc* Tukey analysis. *n* = 4 mice per group.

Immunization with PSNPs-MSP4/5 showed strong T cell and antibody responses, especially following a two-immunization regime, therefore, we next determined the effect of PSNP dose on the resulting immune response. De-escalating doses of PSNPs (2.1 × 10^14^, 3.7 × 10^13^, and 1.7 × 10^13^ PSNP/ml) conjugated to matched levels of MSP4/5 were used to assess the potential for dose-sparing of the vaccine preparation, for both immunogenicity and protection studies. As IFN-γ has been identified as the key protective cytokine (47), the effect of different doses of PSNPs (conjugated to the same amount of MSP4/5) were assessed for their ability to affect the resulting IFN-γ T cell response. De-escalating doses of the PSNPs were compared to CFA/IFA adjuvated formulations for IFN-γ T cell responses following two immunizations. As expected, MSP4/5 in CFA/IFA produced significantly higher T cell responses compared to controls (Figure 3, *p* < 0.05 compared to MSP4/5 alone and naïve). The three doses of PSNPs-MSP4/5 elicited differing IFN-γ T cell responses. Both the highest and lowest PSNP doses (2.1 × 10^14^ and 1.7 × 10^13^ PSNPs/ml-MSP4/5) induced IFN-γ producing T cell responses comparable to CFA/IFA adjuvanted vaccines and both significantly higher than control groups (Figure 3, *p* < 0.0001 2.1 × 10^14^ PSNPs-MSP4/5 compared to MSP4/5 alone, *p* < 0.001 compared to naïve, *p* < 0.001 1.7 × 10^13^ PSNPs-MSP4/5 compared to MSP4/5 alone, *p* < 0.01, compared to naïve). Unexpectedly, both the highest and lowest PSNP doses also induced significantly higher IFN-γ T cell responses compared to the 3.7 × 10^13^ PSNPs-MSP4/5 dose (Figure 3, *p* < 0.01 and *p* < 0.05, respectively), indicating no clear titratable effect of the PSNPs dose.

### Immunization With PSNPs-MSP4/5 Induces Survival Against Blood-Stage Malaria Infection

**Figure 4:**
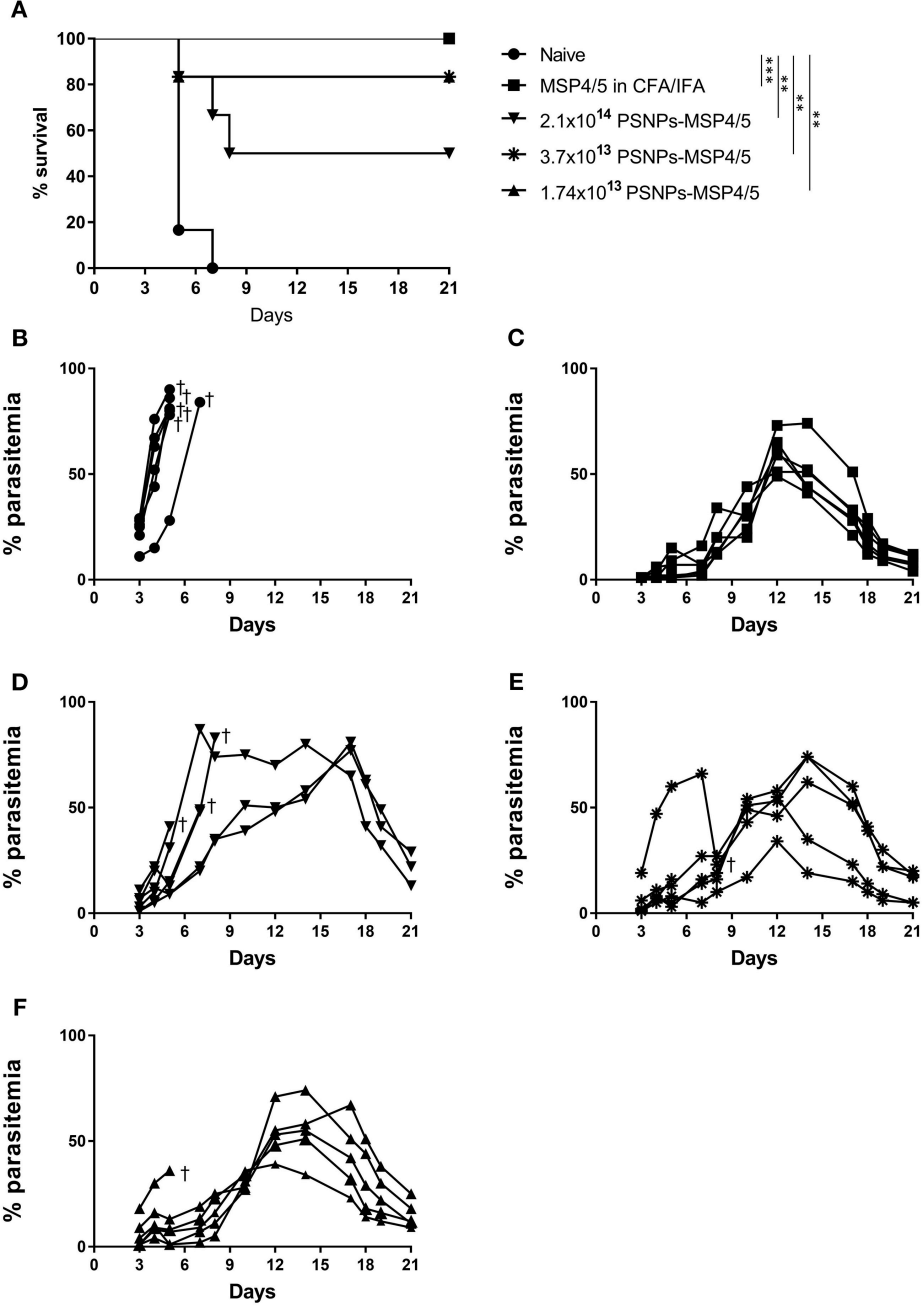
Moderate survival against blood-stage malaria infection following immunization with de-escalating doses of PSNPs compared to CFA/IFA adjuvanted vaccines. BALB/c mice were immunized intradermally at the base of tail (100 μl/immunization) and then a boost 3 weeks later with PBS alone (Naïve), MSP4/5 in CFA (80 μg per mouse, first immunization) or MSP4/5 in IFA (80 μg per mouse, boost immunization) or 3 different doses of PSNPs-MSP4/5 (2.1 × 10^14^, 3.7 × 10^13^, 1.7 × 10^13^ PSNPs/ml). Fourteen days after the last immunization, mice were challenged intraperitoneally with 5 × 10^5^ *P. yoelii* pRBCs and parasitemia levels were monitored starting at 3 days post-challenge. **(A)** Kaplan-Meier survival curve and percentage parasitemias of mice immunized with **(B)** Naïve -∙-, **(C)** MSP4/5 in CFA/IFA -■-, **(D)** 2.1 × 10^14^ PSNPs-MSP4/5 -▼-, **(E)** 3.7 × 10^13^ PSNPs-MSP4/5 -^*^- and **(F)** 1.74 × 10^13^ PSNPs-MSP4/5 -▲-. ^**^p < 0.01, ^***^p < 0.001, with Mantel Cox Log Rank Test. Indicates mouse endpoint. Data shown are from 6 mice per experimental group.

Not only is it important to identify vaccine formulations that elicit strong T cell and antibody responses, they also need to translate into a clinical effect of blood-stage protection and/or enhanced survival. To investigate this, mice were immunized twice, 3 weeks apart with de-escalating doses of PSNPs-MSP4/5 and MSP4/5 in CFA/IFA. Two weeks following immunizations, mice were then challenged with *P. yoelii* parasites and monitored for parasitemia levels and overall survival. Mice reached end point when blood smears showed over 70% parasitemia levels. All groups survived significantly longer than naïve mice (Figure 4A, *p* < 0.001 for MSP4/5 in CFA/IFA and *p* < 0.01 for all doses PSNPs-MSP4/5, compared to naive mice) as shown on the Kaplan-Meyer survival curve. Despite observing different survival curve patterns, there were no significant differences between MSP4/5 in CFA/IFA and the three doses of PSNPs-MSP4/5 up until 21 days post-challenge, indicating these are comparably protective vaccine formulations. In terms of overall survival, the control naïve mice all reached endpoint parasitemia's (Figure 4B). All mice in the MSP3/5 in CFA/IFA group resolved their parasitemia levels 6/6 (Figure 4C), in comparison to 5/6 mice in both the 3.7 × 10^13^ and 1.7 × 10^13^ PSNPs-MSP4/5 groups (Figures 4E,F), and 3/6 mice in the 2.1 × 10^14^ PSNP-MSP4/5 group resolved their parasitemia levels (Figure 4D). This suggests the observed partial protection is not dependent on PSNP dose, regardless of significant differences in immune responses induced.

### Moderate Blood-Stage Malaria Protection Induced by PSNPs-MSP4/5 Is IFN-γ dependent

**Figure 5:**
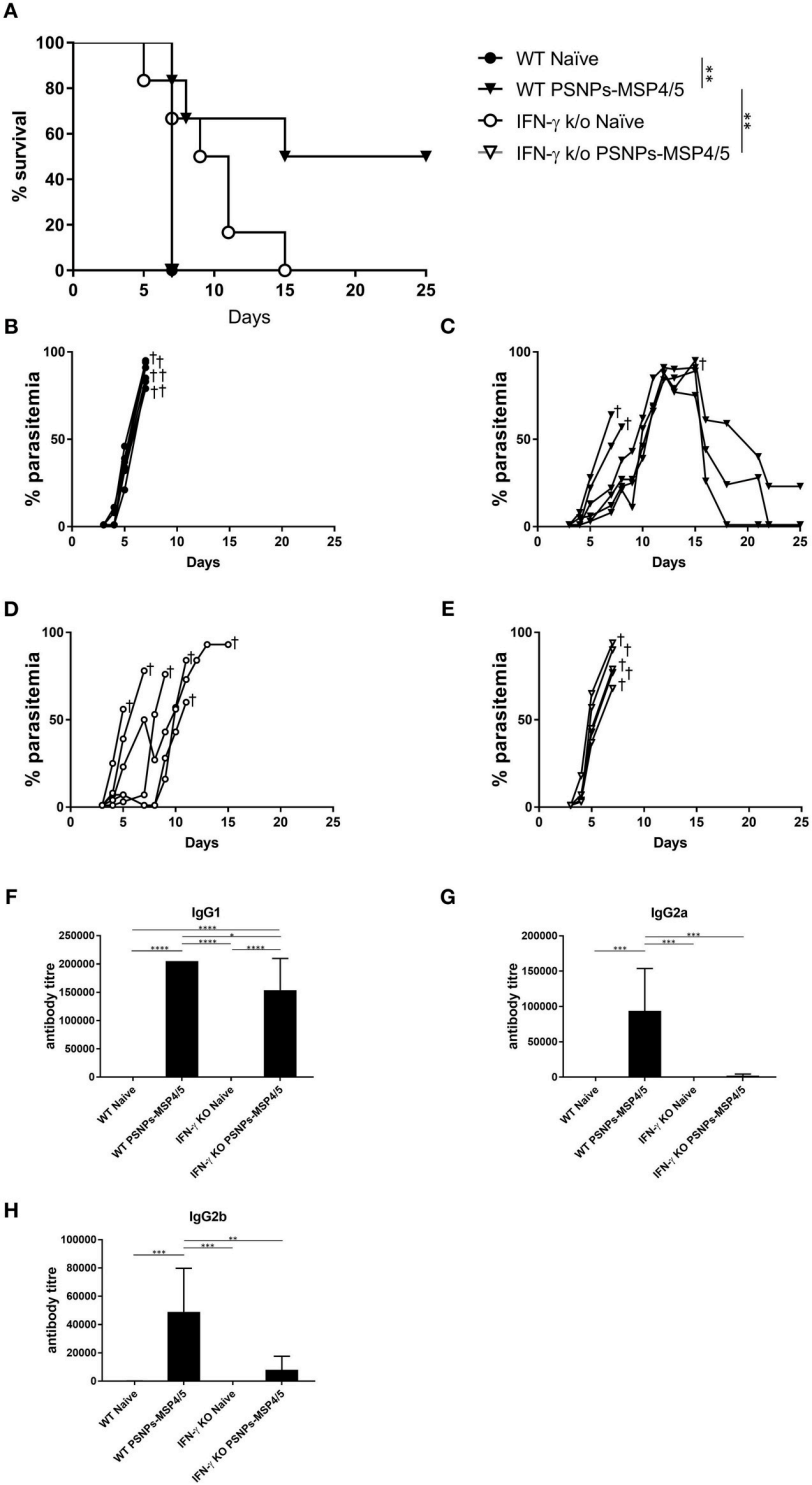
Blood-stage malaria protection induced by immunization with PSNPs-MSP4/5 is IFN-γ dependent. BALB/c WT or IFN-γ KO BALB/c mice were immunized twice intradermally at the base of tail (100 μl/immunization), 14 days apart with PBS alone (Naïve) or PSNPs-MSP4/5 (100 μg/ml MSP4/5). Fourteen days following the boost immunization, mice were challenged intraperitoneally with 5 × 10^4^ pRBCs from *P. yoelii* and survival parasitemia levels were monitored starting at 3 days post-challenge. **(A)** Kaplan-Meier survival curve and parasitemia levels of mice immunized with **(B)** WT Naïve -∙-, **(C)** WT PSNPs-MSP4/5 -▾-, **(D)** IFNγ KO Naïve -○- and **(E)** IFNγ KO PSNPs-MSP4/5 -▽-. Pre-challenge **(F)** IgG1, **(G)** IgG2a, and **(H)** IgG2b anti-MSP4/5 antibody levels are also shown as mean antibody titer ± SD per group. ^*^p < 0.05, ^**^p < 0.01, ^***^p < 0.001, ^****^p < 0.0001 with Mantel Cox Log Rank Test for survival analysis and one-way ANOVA for antibody titer. Indicates mouse endpoint. Data shown are from 5−6 mice per experimental group.

In the above studies, high levels of IFN-γ and IL-4 producing T cells were induced by PSNPs-MSP4/5 vaccine formulations, as well as antibody responses including diverse IgG subclasses. Generally, functional antibodies have been associated with protective responses in humans for blood-stage malaria, with few studies characterizing the T cell response (48, 49). IFN-γ has been reported to be protective in prior *P. yoelii* liver stage murine malaria models (50), and *P. chabaudi* blood-stage infections (51), therefore, we wanted to assess mechanistically the necessity of IFN-γ production in protecting mice from blood-stage malaria immunized with PSNPs-MSP4/5. Wild type (WT) or IFN-γ KO (IFN-γ KO) BALB/c mice were immunized twice, intradermally, 2 weeks apart, with PSNPs-MSP4/5 and 2 weeks following the last immunization, mice were challenged with *P. yoelii* parasites and monitored for survival and parasitemia levels. As expected, naïve WT mice reached parasitemias over 70% (Figures 5A,B). PSNPs-MSP4/5 immunized WT mice were significantly protected in comparison to naive WT mice (Figure 5A, *p* < 0.01), with 3/6 mice protected up to 25 days post-infection and resolving their parasitemia levels (Figures 5A,C). Both the naïve and PSNPs-MSP4/5 experimental groups reached parasitemias above 70% in the IFN-γ KO mice (Figures 5A,D,E). There was a significant difference in survival between the PSNPs-MSP4/5 immunized WT mice compared to PSNPs-MSP4/5 immunized IFN-γ KO mice (Figure 5A, *p* < 0.01) suggesting protection may be IFN-γ dependent. There was also a significant difference between the immunized WT mice compared to naive WT mice (Figure 5A, *p* < 0.01), but no significant difference between the immunized and naïve IFN-γ KO mice. Some naive IFN-γ KO mice survived for marginally longer than PSNPs-MSP4/5 immunized mice, though this result was not significant.

Furthermore, antibody responses pre-challenge demonstrated high levels of IgG1 antibodies in PSNPs-MSP4/5 immunized mice in both the WT and IFN-γ KO mice, with both inducing antibody titres significantly higher than WT naïve mice and IFN-γ KO naïve mice (Figure 5F, *p* < 0.0001). PSNPs-MSP4/5 immunized WT mice also induced significantly higher IgG1 antibody levels compared to PSNPs-MSP4/5 immunized IFN-γ KO mice (Figure 5F, *p* < 0.05). IgG2a and IgG2b induction was impaired in IFN-γ KO mice but not in WT mice when immunized with PSNPs-MSP4/5. IgG2a antibody levels elicited by PSNPs-MSP4/5 in WT mice were significantly higher than WT naïve mice and both IFN-γ KO naïve and immunized mice (Figure 5G, *p* < 0.001). IgG2b antibodies were also significantly higher in the WT PSNPs-MSP4/5 immunized mice compared to WT naïve and IFN-γ KO naïve mice (Figure 5H, *p* < 0.001) and PSNPs-MSP4/5 immunized IFN-γ KO mice (Figure 5H, *p* < 0.01). These results may be explained when considering IgG1 antibodies are induced by IL-4 and associated with Th2 responses and IgG2 antibodies are associated with Th1 responses a and dependent on IFN-γ.

## Discussion

The present study shows immune responses induced by a biocompatible nanoparticle carrier vaccine approach can induce immunity and moderate protection against blood-stage malaria comparable to the “gold standard” experimental adjuvant CFA. This finding adds to the repertoire of possible adjuvants and vaccine approaches in development, by showing that delivering a classical pro-inflammatory danger signal is not necessary to induce immune responses of sufficient magnitude, or of the type, necessary to protect against blood-stage malaria infection. We note, however, that associations of immune responses with protection do not necessarily imply causality. Nonetheless, the above results support the MSP4 and MSP5 families as candidate antigens for vaccine development when used with an appropriate delivery system.

PSNPs have repeatedly shown, in a range of disease models, to be non-inflammatory and capable of inducing robust immune responses without the addition of T helper epitopes or additional stimuli (31, 37, 42, 44, 52). Side by side comparisons of immunogenicity elicited by PSNPs-MSP4/5 showed them to be capable of producing superior cellular responses to MSP4/5 in Alum, inducing balanced Th1 and Th2 responses, as well as comparable responses to MSP4/5 in CFA/IFA vaccine formulations, the latter representing a gold standard level of immune responses in experimental models. Similar results were obtained with MSP4/5 with an alternative production system in *Saccharomyces cerevisiae* (unpublished).

Antigen specific IFN-γ production by T cells was predominantly measured in the present study, though as whole splenocytes were used in the assays there is a possibility that IFN-γ production from other cell types (i.e. NK cells) may also be measured, but unlikely to this protein antigen. The cells that respond in ELISpot assays are predominantly T cells, as shown by depletion assays (53–55). Dose-sparing of the PSNP formulations unexpectedly showed that the middle dose induced significantly lower IFN-γ producing T cell responses, compared to the highest and lowest PSNP doses. Therefore, it will be useful in future studies to explore the dose dependency of other types of immune responses.

PSNPs-MSP4/5 formulations showed substantial improvement over MSP4/5 in Alum formulations in the elicitation of specific antibody subclasses such as IgG2a and IgG2b, but not total Ig or IgG1. Although the antibody titers induced by PSNPs-MSP4/5 vaccines were lower than the “gold standard” MSP4/5 in CFA/IFA, PSNPs-MSP4/5 vaccines induced moderate protective immunity compared to CFA/IFA adjuvanted formulations. Though only CFA/IFA adjuvanted formulations achieved complete protection, it has been noted in this model that is difficult to observe complete protection (22, 24, 46). Surprisingly, there were no significant differences in the protection observed between the different PSNP doses, however there was a slightly lower survival rate in the group immunized with the highest dose of PSNPs. Overall, the partial protection observed with PSNPs in this model shows promise for current and future nanoparticle delivery platforms for blood-stage malaria.

Studying the mechanism of the protective immunity against malaria induced by PSNPs-MSP4/5, using knockout mice, demonstrated protection to be IFN-γ dependent. This is consistent with reports in the literature suggesting IFN-γ is the critical mediator of protection in erythrocytic stage infections (47), and for other strains of blood-stage Plasmodium infection (51), though the exact mechanism of protection has yet to be defined. Our results show a change in IgG subclasses elicited by the vaccine, with sustained IgG1 (Th2 associated) production but significantly decreased anti-MSP4/5 levels of IgG2a and IgG2b (Th1 associated) compared to non-immunized controls. These results indicate that the induction of IgG1 alone is not protective in this model (56), as the IFN-γ KO mice are still able to produce IgG1 but are not protected. Previous studies have shown that high levels of both IgG1 and IgG2a are required to mediate protection in the *P. yoelii* model (57, 58) however both Hirunpetcharat et al. (59) and Oakley et al. (60) showed that IgG1 alone correlates with protection against *P. yoelii* challenge (59, 60). In contrast, Adame-Gallegos et al. (61) showed that IgG1 did not mediate protection against *P. yoelii* (61).

One interesting finding in the knock out experiment was that the IFN-γ KO naïve mice had slightly better survival that the immunized KO mice (though this was not significant). Whilst it is possible that this is due to the potency of the parasites themselves, it may also be due to the differences in vaccine induced responses. As these mice do not have any vaccine induced responses, compared with the PSNPs vaccinated mice, there may be other mechanisms compensating for IFN-γ and contributing to the marginally increased survival. A potential cell type that has been identified as having a key role in clearing early parasitemia's are γδ T cells (62). It is possible that without the expansion of αβ T cells as would be expected in vaccinated animals, γδ T cells may expand instead, as well as there being a potential difference in cytokine profiles, especially from innate cells (63). Though it cannot be proven for the present study, the above hypothesis would be of interest to investigate further in this model in future studies. Indeed, in future studies it would be preferable to explore other immune responses induced by these PSNPs vaccines in the context of blood-stage malaria, including investigating other cytokines (i.e., TNF, IL-5, IL-17) in addition to the hallmark Th1 and Th2 cytokines IFN-γ and IL-4, respectively. Likewise, it would be beneficial to examine immune cell types such as B cells, γδ T cells, Th17, Tfh, Tr1, and T regulatory cells, as well as individual subsets within these populations to explore the different effector functions and cytokine profiles within cell subsets.

*Plasmodium falciparum* derived MSP4 and MSP5 are closely related to the MSP4/5 *P. yoelli* antigen used in this murine model of malaria. Their attractiveness as an antigen for target development came from the finding that they, and particularly MSP5, are largely conserved across malaria species, and show little polymorphism when analyzing within any single species of malaria parasites (10, 15). However, enthusiasm declined when it was shown that naturally found antibodies against MSP4 or MSP5 had little neutralizing capacity in *in vitro* red cell parasite growth inhibitory assays (GIA). Other MSP antigens were also shown to be associated with reduced incidence of malaria (64), and have been more widely studied. Although recent studies have shown association of both MSP4 and MSP5 antigens with decreased cases of malaria, specifically severe malaria (18, 19). The present study reinforces the contention that it is possible to deliver vaccine induced protection against malaria blood-stage, focusing on the induction of robust Th1 immunity, and not necessarily particular antibody functions. Of note is the fact that >60% humans naturally exposed to either acute *P. falciparum* (63%) or *P. vivax* (67%) malaria infection show IFN-γ responses to PfMSP5, suggesting it is (1) immunogenic in humans, (2) has the potential to be boosted by natural infection, and (3) is potentially a cross-reactive antigen between the two main stains of malaria found in many malaria endemic regions of the globe, including Asia (20). In agreement with previous studies, the current study suggests it is possible to design vaccines capable of inducing such potentially useful T cell responses. Moreover, we show it is possible to do so using carriers and adjuvants that do not need to deliver classical pro-inflammatory signals, a potentially important consideration when vaccinating infants in malaria endemic areas. Given our positive findings with PSNPs-MSP4/5, this supports the usefulness of further exploring other diverse nanoparticles as they are developed, as carriers for MSP antigens, beyond PSNPs. Conventional adjuvants carry the risk of damaging inflammatory responses, as well as data emerging from the literature suggesting that non-live adjuvanted vaccines may be directly associated with unhelpful non-specific effects (65). Overall, we hope the present study will spur further research into non-inflammatory vaccine carriers and adjuvants, as well as the potential for Th1 associated vaccine induced immunity in blood-stage malaria, which may help us re-examine from an additional perspective the nature of the antigens for inclusion in malaria vaccines.

## Author Contributions

MP designed and supervised all experiments and analyzed and interpreted all the data. KW designed and performed some of the experiments and analyzed some of the data. DP designed and performed some of the experiments and analyzed some of the data. JH and CM performed some of the experiments. SX designed and supervised some of the experiments and analyzed some of the data. RC planned some of the experiments. MP, KW, and DP wrote the manuscript and all authors contributed segments and editing to the manuscript preparation.

### Conflict of Interest Statement

Some of the experiments in Figures 3 and 4 were funded by Panvax Pty Ltd. JH worked for Panvax during this period. MP was a Director of Panvax 2001-2007 and PX Biosolutions Pty Ltd 2007-2016. The remaining authors declare that the research was conducted in the absence of any commercial or financial relationships that could be construed as a potential conflict of interest.
